# Supplementary material for: Long-Term SMN- and Ncald-ASO Combinatorial Therapy in SMA Mice and NCALD-ASO Treatment in hiPSC-Derived Motor Neurons Show Protective Effects
Source: Int J Mol Sci. 2023 Feb 20;24(4):4198. doi: 10.3390/ijms24044198 (PMC9961752; doi:10.3390/ijms24044198)
Supplement: Supplementary file 1 [file ijms-24-04198-s001.zip › ijms-2218545-supplementary.pdf]

# Supplementary Information

# Long-term *SMN*- and *Ncald*-ASO combinatorial therapy in SMA mice and *NCALD*-ASO treatment in hiPSC-derived motor neurons show protective effects

Anixa Muiños-Bühl [1,2], Roman Rombo [1,2], Karen K. Ling [3], Eleonora Zilio [1,2], Frank Rigo [3], C. Frank Bennett [3], and Brunhilde Wirth [1,2,4,*]

[1] Institute of Human Genetics, University of Cologne, Cologne, Germany
[2] Center for Molecular Medicine Cologne, University of Cologne, Cologne, Germany
[3] IONIS Pharmaceuticals, Carlsbad, California, USA
[4] Center for Rare Diseases Cologne, University Hospital of Cologne, Cologne, Germany
***** Correspondence: brunhilde.wirth@uk-koeln.de

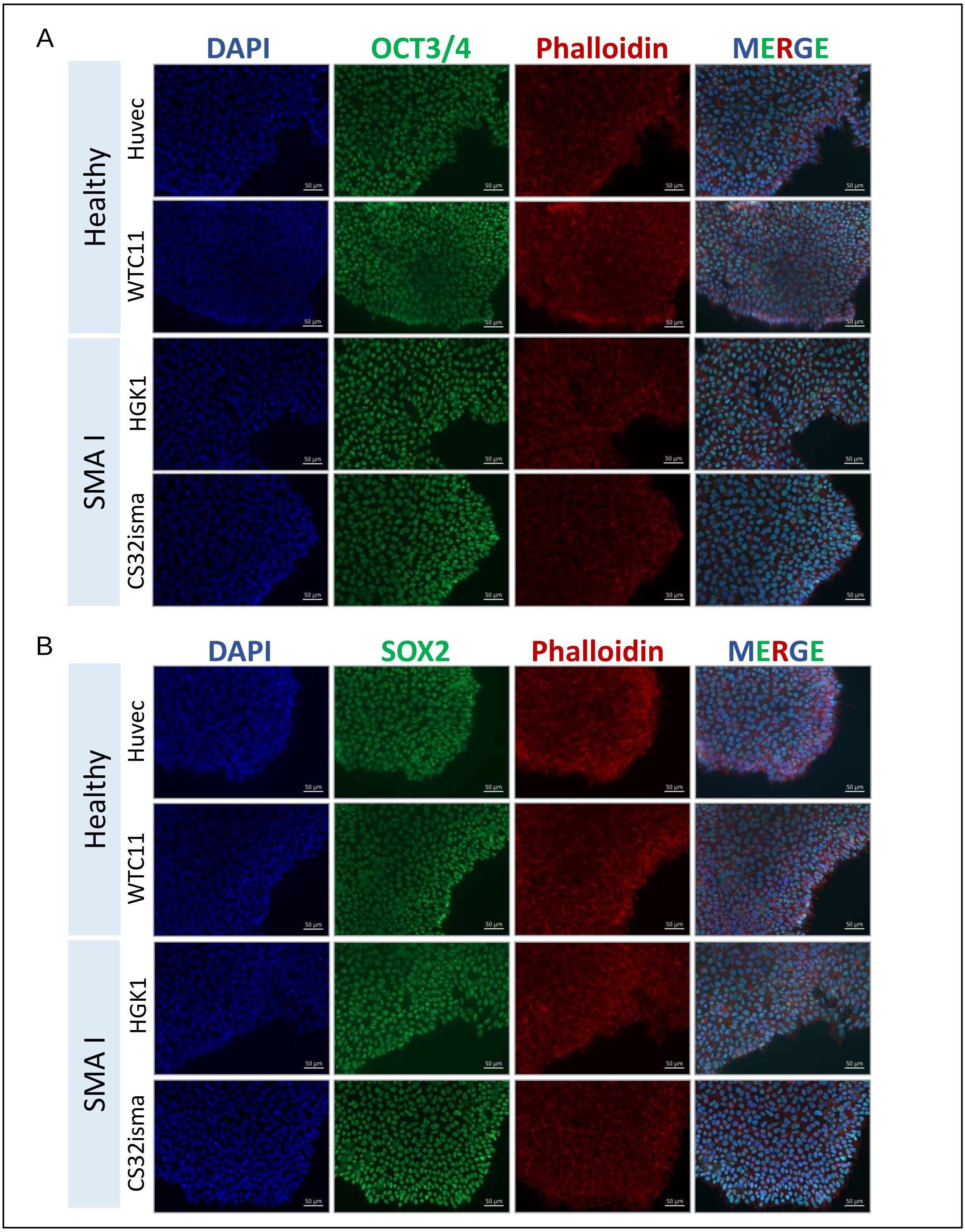


**Figure S1. hiPSCs staining of pluripotency markers OCT3/4 and SOX2.**

(**A-B**) Representative pictures of typical flat colony morphology in all hiPSC lines. Cells were immunostained for the pluripotency marker (**A**) OCT 3/4 (green) and (**B**) SOX2 marker (green). Nuclei were counterstained with DAPI (blue) and actin with Phalloidin (red). Scale bar 100 µm.

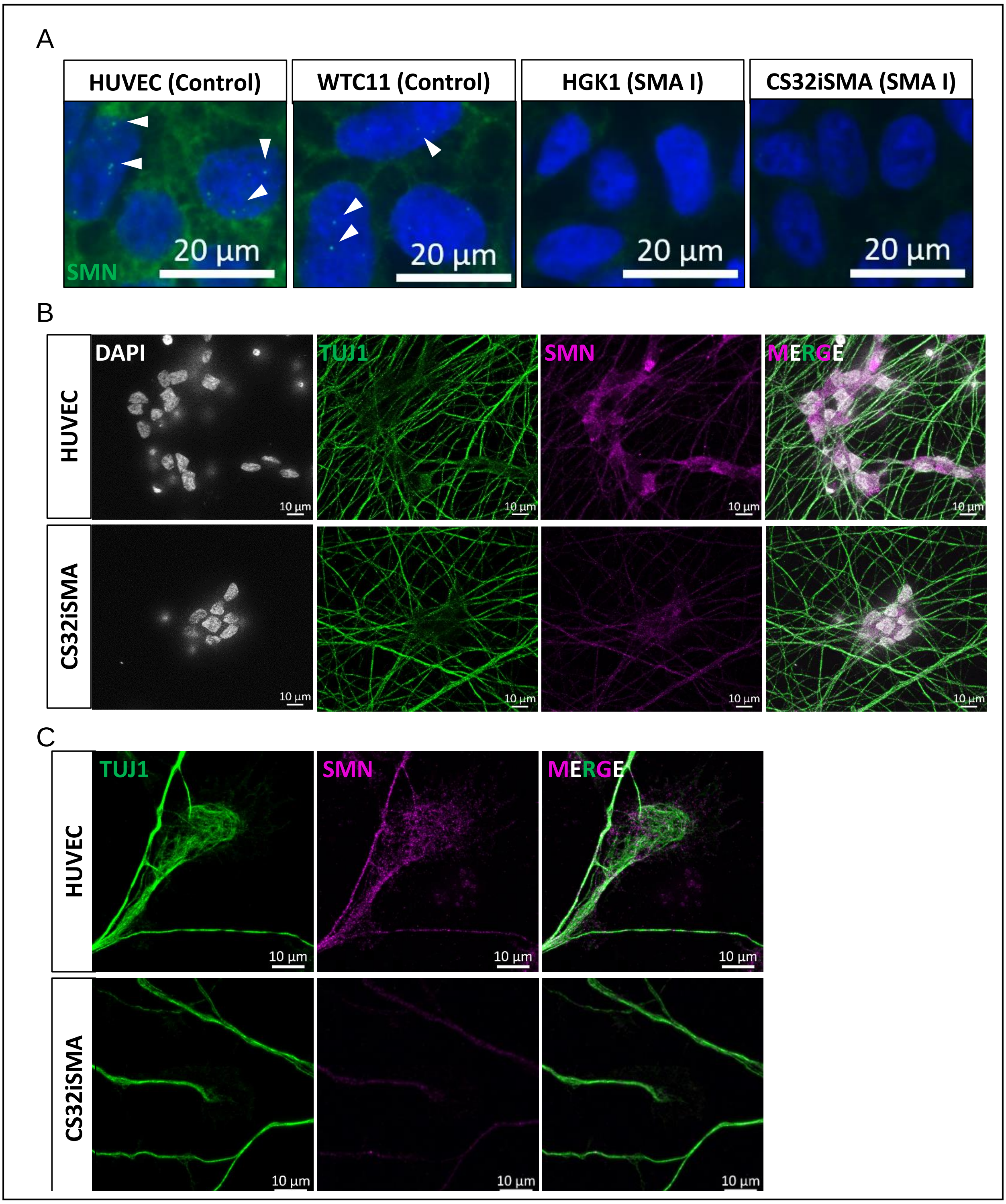


**Figure S2. SMN protein in hiPSCs and hiPSC derived MNs.**

(**A**) SMN staining (green) showed the characteristic dots in the nuclei (blue, DAPI) that represents the gems (marked with white arrows) in both HUVEC and WTC11 healthy lines, and presence of SMN in the cytosol. SMN gems were not observed in SMA type I (HGK1 and CS32iSMA) and there was a visible reduction of SMN staining in the cytosol. Scale bar 20 µm. (**B-C**) SMN staining (magenta) of control and SMA type I MNs derived from hiPSCs. (**B**) SMN (magenta) localizes at the nucleus (white, DAPI), cytoplasm and axons (green, TUJ1) of control MNs, whereas a strong reduction of the signal was observed in SMA type I MNs. Scale bar 10 µm. (**C**) SMN (magenta) accumulates at the presynaptic terminal, the growth cone. Axons are counter stained with TUJ1 (green), a microtubule marker. Scale bar 10 µm.

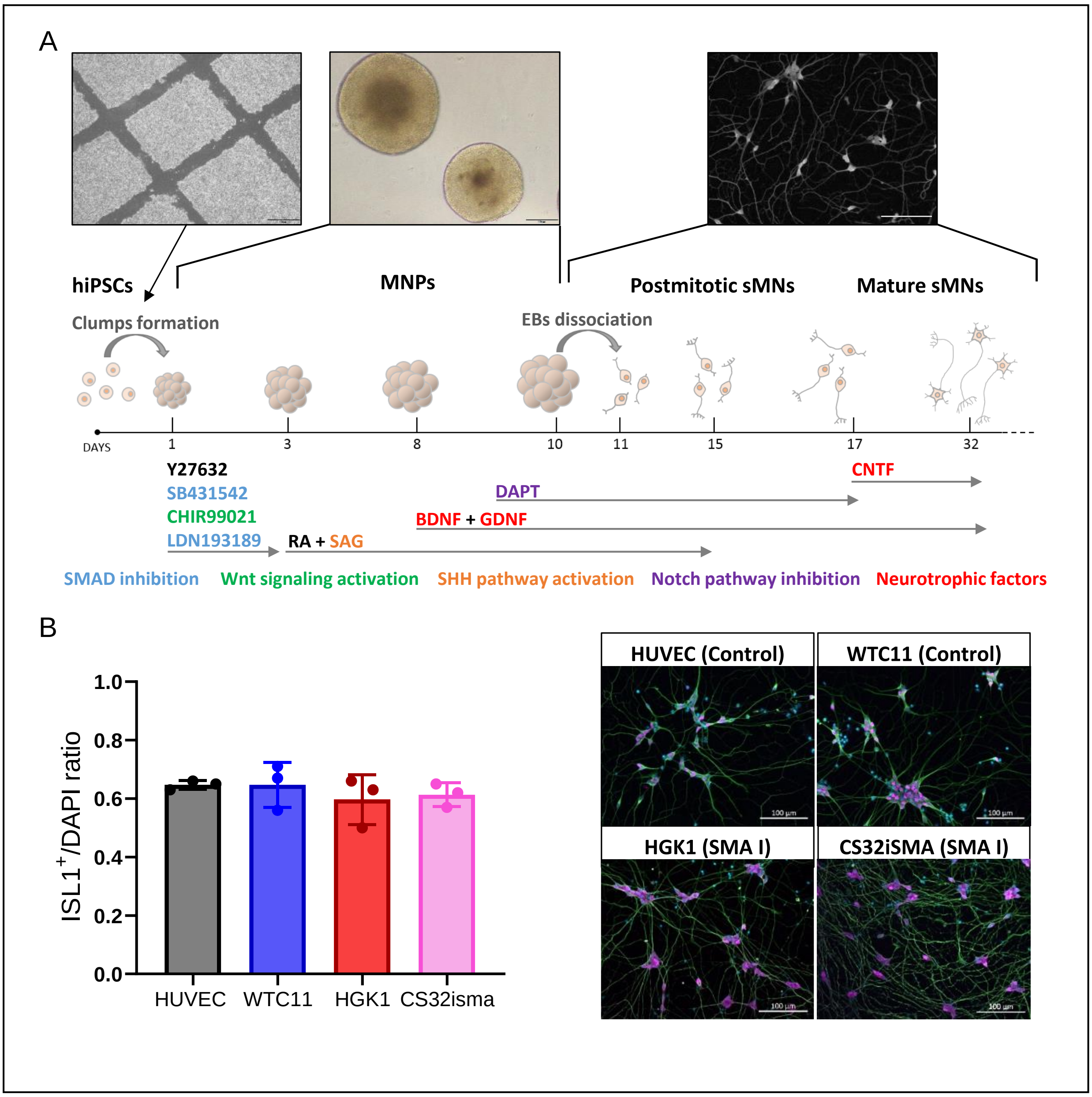


**Figure S3. MN differentiation protocol and efficiency.**

(**A**) Left image shows the similar-sized squares manually made to help with the formation of the clumps (scale bar 500 µm). Central image shows aspheric embryoid bodies from the WTC11 hiPSC line at day 7 (scale bar 200 µm). Right image is a representative picture of a MN culture derived from control HUVEC hiPSCs at d15 of the differentiation (scale bar 100 µm). Above of the gray arrows are the abbreviation of the cytokines used in the respective days during the differentiation, and below, following the color code, their role in the differentiation. MNPs refers to MN progenitors. (**B**) MN specification was determined by the presence of ISL1 positive staining (magenta), axons were counterstained with β-III-tubulin (green), and nuclei with DAPI (blue) at day 15 of the differentiation. Scale bar: 100 µm for all images. Ordinary one-way ANOVA with Tukey posthoc test for multiple comparisons. Error bars represent ± SD. Each dot represents the average ratio of $ISL1^{+}$/DAPI of each independent differentiation, N=3 for all hiPSC lines. Total number of cells counted per line is: HUVEC = 1461, WTC11 = 1534, HGK1 =1466, CS32iSMA = 1289.

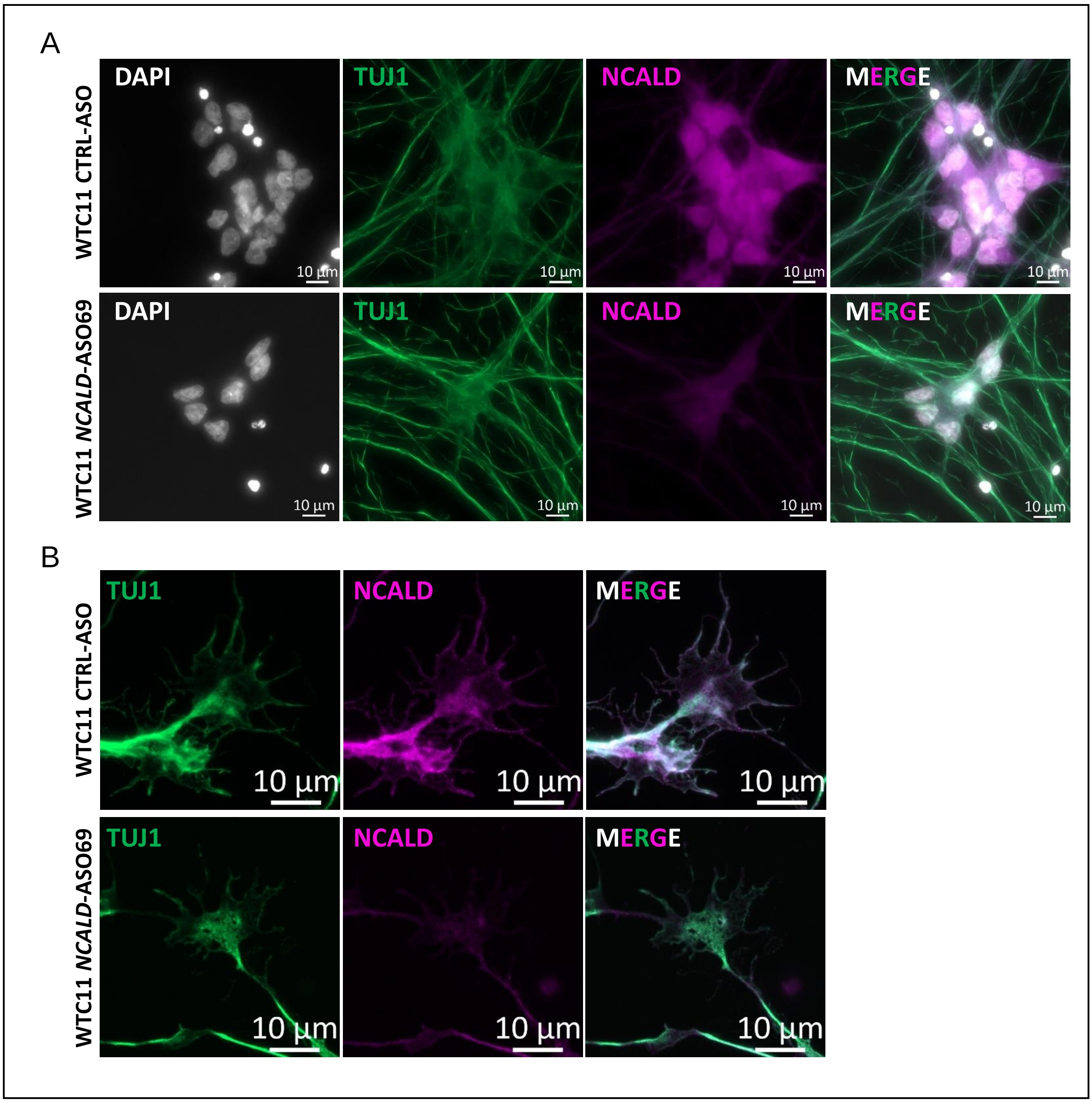


**Figure S4. NCALD reduction in MNs upon treatment with *NCALD*-ASO69.**

(**A-B**) NCALD staining (magenta) of control MNs derived from WTC11 hiPSCs, treated with 60 nM CTRL-ASO or *NCALD*-ASO69 at day 13, immunostaining performed at day 20 of the differentiation. NCALD localizes at A) the cytosol of MNs and B) growth cones and is markedly reduced upon *NCALD*-ASO69 treatment. Nuclei were counter stained DAPI (white) and axons are stained with TUJ1 (green) a microtubule marker. Scale bar 10 µm.

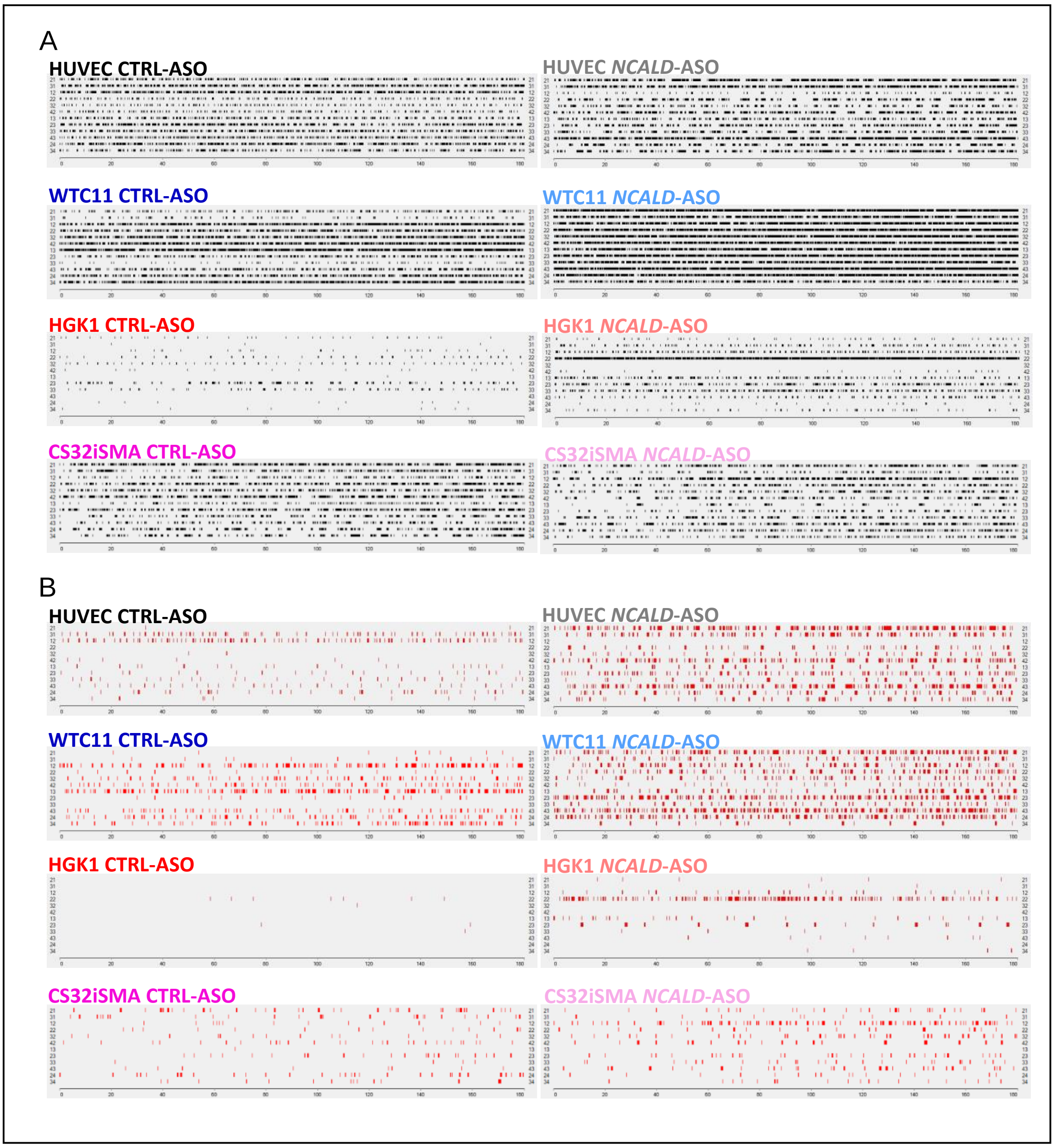


**Figure S5. Spikes and burst of healthy and SMA hiPSC derived MNs upon *NCALD*-ASO69 treatment.**

(**A-B**) MEA analysis at day 20 of CTRL-ASO or *NCALD*-ASO69 treated MNs. Each image represents (**A**) spikes (black) and (**B**) burst (red) detected in 1 well, recorded for 3 minutes. Each line represents the activity detected by one electrode (in total 12 electrodes per well). In total, three independent experiments from three independent MN differentiations were recorded.
